# Successful application of anthranilic diamides in preventing small hive beetle (Coleoptera: Nitidulidae) infestation in honey bee (Hymenoptera: Apidae) colonies

**DOI:** 10.1093/jisesa/iead096

**Published:** 2023-12-06

**Authors:** Ethan J Hackmeyer, Tyler J Washburn, Keith S Delaplane, Lewis J Bartlett

**Affiliations:** Center for the Ecology of Infectious Diseases, Odum School of Ecology, University of Georgia, Athens, GA 30602, USA; Department of Microbiology, Franklin College of Arts and Sciences, University of Georgia, Athens, GA 30602, USA; Department of Entomology, College of Agricultural and Environmental Sciences, University of Georgia, Athens, GA 30602, USA; Center for the Ecology of Infectious Diseases, Odum School of Ecology, University of Georgia, Athens, GA 30602, USA; Department of Entomology, College of Agricultural and Environmental Sciences, University of Georgia, Athens, GA 30602, USA

**Keywords:** pest control, feeding behavior, IPM, pesticide development/analysis

## Abstract

The nest-scavenging beetle *Aethina tumida* remains a persistent problem for beekeepers in parts of the Southeast United States, where warm wet soils allow beetle populations to grow rapidly and overwhelm colonies, especially during the summer dearth. Furthermore, small hive beetle infestation prevents beekeepers from easily provisioning colonies with additional pollen or protein feed (patties), preventing holistic management of honey bee health via improved nutrition, and reducing the economic potential of package and nucleus colony rearing in the Southeast. Here, we demonstrate using both in vitro laboratory trials and a small in vivo field trial that the differential specificity of anthranilic diamide insecticides (specifically, chlorantraniliprole) between bees and beetles allows for the control and prevention of small hive beetle infestation in honey bee colonies even when feeding with large patties. Honey bees show orders of magnitude higher tolerance to chlorantraniliprole compared to small hive beetles, opening new avenues for improving bee health including during spring splits and throughout the summer.

## Introduction

Small hive beetles, *Aethina tumida* Murray, are a prominent honey bee (*Apis mellifera* L.) pest across much of the United States, first invading the continental United States via the Southeast in the 1990s (Elzen et al. 1999, Roth et al. 2022), as well as challenging beekeeping in other regions such as the Northeast coast of Australia and parts of South Africa (Cuthbertson et al. 2013). European beekeeping remains on high alert for the expansion of *A. tumida* into the Mediterranean Basin and beyond (EFSA 2015). *Aethina tumida* are an established destructive pest in the Southeast of the United States, and anecdotally kill colonies if placed in shade or following tropical storms. Whilst mechanical and biocontrol techniques to control *A. tumida* are widely understood and used by beekeepers, these efforts have failed to fully control this burdensome honey bee parasite (Buchholz et al. 2011, Bartlett 2022, Roth et al. 2022).

A consequence of *A. tumida*’s parasitism is a widespread reluctance amongst beekeepers in the Southeast to undertake ad-libitum supplementary pollen (or pollen substitute) feeding as part of routine colony management. While feeding sugar solutions is a common practice to aid in colony provision, pollen supplementation is rare, in part due to risks of severe *A. tumida* infestation. This is despite widespread evidence that polyfloral pollen is a critical component of honey bee health, for instance in reducing infectious pathogen burdens (Alaux et al. 2010) including viruses (DeGrandi-Hoffman and Chen 2015, Dolezal et al. 2019), which are a pernicious contributor to honey bee decline. While substitute, nonpollen protein feeds typically are shown to be inferior to pollen (DeGrandi-Hoffman et al. 2010, 2016, Noordyke and Ellis 2021), when compared with unfed controls these substitute feeds improve a range of colony health metrics including infection outcomes (DeGrandi-Hoffman et al. 2010). Even amongst natural pollen, there is significant variation in its value in improving viral infection outcomes (Dolezal et al. 2019, Walton et al. 2021). Unfortunately, many studies on this topic opt not to include unfed control colonies when comparing pollens and pollen substitutes, and additionally often pollen-trap colonies preventing normal foraging, limiting how informative they are for making management decisions (e.g., DeGrandi-Hoffman et al. 2016). In the Southeast, it can be difficult to run these experiments due to the challenge of beetle infestation.

With increasing challenges in reducing the burden of other parasites and viruses in honey bee colonies (Bartlett 2022), the indirect role of nutrition in helping bolster honey bee immunity is therefore increasingly crucial to the overall health of the industry (Dolezal and Toth 2018). The overlap between severe *A. tumida* infestation occurrence and the highly destructive ectoparasitic mite *Varroa destructor* is not coincidental. The weather patterns, especially sustained high temperatures and humidity (Bernier et al. 2014), that promote severe *A. tumida* infestation also maintain year-long brood area in colonies for *V. destructor* reproduction (Berry et al. 2022) and also preclude beekeepers from using certain acaricides (namely, the highly effective organic acaricide formic acid). This *A. tumida*, *V. destructor* syndemic is currently unanswered (Bartlett 2022), with heightened viral titers in bees caused by extended *V. destructor* reproduction seasons exacerbated by inhibited supplementary feeding of pollen or other alternatives due to fears of attracting *A. tumida*.

As far back as the 1960s, the American beekeeping industry had identified *A. tumida* as a potential threat to US beekeeping. Decades before their arrival in the United States, Caron (1978) had stated that ‘*One can only hope the beetle will not be transported to other beekeeping areas*’. Despite their long-identified threat to American beekeeping, very few acute controls have been labeled or licensed for in-hive use (Kanga and Somorin 2012, Roth et al. 2022). Furthermore, no acute control has been licensed for “safeguarding” supplementary pollen or protein substitutes from inviting severe *A. tumida* infestation. Therefore, there remains a high demand amongst beekeepers for products which are safe for consumption by honey bees but toxic to small hive beetles. Of note is the recent increase in reports of beekeepers struggling to manage colony health in the face of small hive beetle outbreaks, putatively linked to unusual seasonal weather patterns and the ever-looming threat of climate change (Cornelissen et al. 2019).

In this manuscript, we report on the suitability of the anthranilic diamide chlorantraniliprole as a coleoptericide for deployment in honey bee colonies, including as a deterrent/toxin suitable for mixing into protein substitutes fed to colonies. This work was inspired by the novel control of turf lawn beetle grubs using chlorantraniliprole as an environmentally safer alternative to the previously used neonicotinoid, clothianidin. Specifically, Larson et al. (2013, 2014) demonstrated that chlorantraniliprole has no detectable negative effects on bumblebee (*Bombus impatiens*) colony growth, mortality, or queen production. Further, EPA registration documentation for chlorantraniliprole as a lawn-treatment shows extremely low toxicity to *Apis mellifera*—with an LD50 in excess of >0.1 mg/bee, being more than 2,000× less toxic than a comparable neonicotinoid, clothianidin (EPA 2008). Chlorantraniliprole has a specific mode of action: it stimulates ryanodine receptors, causing calcium store release in insect muscle, eventually resulting in lethal paralysis (Cordova et al. 2006). It is hypothesized that molecular differences in the sarcoplasmic reticulum calcium-release pathway between the Hymenoptera and the Coleoptera lead to its atoxicity in Hymenoptera. The degree of atoxicity of chlorantraniliprole has been widely explored in honey bees; and while Kadala et al. (2019) documented some sublethal effects and an aberrantly low LD_50_ (Kadala et al. 2019), numerous other works have shown a comparative lack of toxicity in *A. mellifera* exposed to chlorantraniliprole (Lahm et al. 2007, Williams et al. 2020, Walker et al. 2022). We also began preliminary testing of another closely related anthranilic diamide, flubendiamide, but focused efforts on chlorantraniliprole at the recommendation of the developers of flubendiamide (Schmehl, D.; Bayer Crop Sciences, pers. comm.) as it is currently not registered for use in the United States. Chlorantraniliprole is however registered, widely used, and consequently, better studied for this prospective use.

Specifically, the toxicity of chlorantraniliprole and its movement through the honey bee colony has been well documented by Ricke et al. (2021), due to its use in almond orchards during honey bee colony deployment for pollination. While chlorantraniliprole shows toxicity when co-applied with certain fungicides (Wade et al. 2019), in isolation it shows no significant effect on honey bees when dosed at field-effective concentrations (Walker et al. 2022). Additionally, honeybees appear capable of buffering developing larvae from exposure to chlorantraniliprole even when fed in pollen (Ricke et al. 2021).

Here, we use in vitro dose–response assays of adult honey bees and adult small hive beetles to characterize the difference in toxicity of chlorantraniliprole to the 2 insects. We also establish minimum preventative doses of chlorantraniliprole in protein supplements intended for honey bees which inhibit the growth of small hive beetles in that media. Finally, we document a proof-of-concept experiment where control protein supplements and treated protein supplements were placed in honey bee colonies for consumption, and the resulting degree of small hive beetle infestation.

## Materials and Methods

### Preliminary Experiments

Preliminary tests were undertaken on small hive beetle larvae collected from infested frames of an absconded colony. Wandering stage larvae were separated into 24 cohorts of 20 individuals (*n* = 480) and provided with 1 ml of honey solution (2:3 honey:water) in standard petri dishes. Larvae were given 3 h to acclimate in a dark incubator at 30 °C. Commercial chlorantraniliprole lawn pellets (GrubEx, Scotts) were agitated in water for 90 min at 30 °C (50 g GrubEx in 450 ml water), yielding a saturated aqueous solution containing a unknown concentration of chlorantraniliprole. After the acclimation period cohorts were split into halves, with 10 individuals of each cohort placed in a new petri dish marked for treatment and the remaining 10 individuals from each cohort placed in new petri dish marked for control. Using fine-mist spray bottles, treatment plates and larvae therein were doused evenly with 1.5 ml chlorantraniliprole lawn pellet solution, while control plates were doused with 1.5-ml water. All plates were then immediately placed back into the 30 °C incubator. After 2 days, all plates were removed from the incubator and survivorship recorded.

Testing on chlorantraniliprole contact effectiveness was also conducted on small hive beetle adults. Adult beetles were placed in petri dishes containing a 1″ × 1″ square of absorbent shop towel soaked with 1 ml of test or control solution. Test solutions included a control solution, supplied to 6 plates of 8 individuals (1:1 sucrose solution by mass mixed 5:1 with water by volume), a methanol-positive control solution supplied to 7 plates of 8 individuals (1:1 sucrose solution by mass mixed 5:1 with methanol by volume), and 2 chlorantraniliprole treatment solutions each supplied to 7 plates of 8 individuals each, following the same mixture protocol as the methanol-positive control with the addition of chlorantraniliprole to establish final concentrations of 5 and 10 μg/ml chlorantraniliprole. All plates were incubated in the dark at 30 °C for 72 h before observation for mortality.

### Laboratory Assays

Oral toxicity trials using adult *Apis mellifera* used typical honey bee toxicology cages, which were modified from food-grade plastic with 2 openings to insert sugar solution feeders. Feeders were modified veterinary-grade luer-slip syringes (ThermoFisher, USA). Mortality assay cages used two 3 ml feeders filled with exactly 3 ml each of test solution suspended from the top of the cage. All assay cages were maintained in a shared dark incubator at 30 °C and 70% relative humidity. Adult honey bees were collected directly into mortality cages in the field the brood frames of healthy, queenright colonies during the daytime. Feed solutions were inserted into cages once sample cohorts had been brought back to the laboratory. Cages only ever contained adults from a single colony, and for any given assay, each colony contributed an equal number of cages to each treatment dose (5 colonies of unrelated locally mated queens). Mortality assays ran for 48 h; upon removal from the incubator, any cages with no remaining sugar solution were flagged for starvation; this only occurred in one trial and all other trials operated with ad-libitum feeding. The number of dead, immobile, and/or alive bees were counted at both the 24 and 48 h marks, after which all cohorts were euthanized by freezing, and the total number of bees counted exactly by hand. Oral test solutions always contained the same concentration of sucrose, whereby a 1:1 sucrose:water solution by mass was made fresh each day and then diluted down with equal volume water and/or methanol. Oral toxicity assays included methanol positive and methanol negative controls, alongside test pesticide solutions. A subset of assays also included a positive control using dimethoate at a concentration 10 μg/ml. Assay one examined the toxicity of chlorantraniliprole at concentrations of 0, 1.25, 2.50, 5.00, and 10.00 μg/ml; assay 2 examined the toxicity of chlorantraniliprole at concentrations of 0, 5, 15, 65, and 250 μg/ml; and assay 3 examined the toxicity of flubendiamide also at concentrations 0, 5, 15, 65, and 250 μg/ml.

Toxicity testing of chlorantraniliprole on adult small hive beetles used wild-caught small hive beetle adults collected by mouth aspiration from colonies in managed apiaries. Adults from the field were given 3 days to acclimate in large containers with ad-libitum feed mix and maintained in a shared dark incubator at 30 °C and 70% relative humidity. Our standard feed mixtures used a 4:2:1 by volume ratio of BeePro Pollen Substitute (Mann Lake), corbicular pollen collected via pollen trap from UGA apiaries, and honey extracted from UGA-managed colonies (nonexperimental). Test feed mixtures were created to introduce controlled concentrations of chlorantraniliprole. 50 mg of chlorantraniliprole was suspended in 50 ml of glycerol, which was then thoroughly incorporated into 100 g of the control feed mixture, yielding a “stock mixture” concentration of 0.3 mg chlorantraniliprole per 1 g feed. Trial feed mixtures with chlorantraniliprole concentrations of 100 μg, 10 μg, 1 μg, and 0 μg/g were achieved via proportional mixing of this high-concentration stock mixture with uncontaminated feed mixtures, and additional glycerol where required. All feed mixtures ultimately contained the same amount of protein supplement, pollen, honey, and glycerol—only suspended chlorantraniliprole concentrations varied. Following the 3-day acclimation period, adult small hive beetles were randomly assigned across 32 new cages, with 8 cages assigned to each treatment (100 μg, 10 μg, 1 μg, 0 μg/g) with an excess of test feed mixture provided in each cage. All cages contained at least 10 individuals, and no more than 12. Mortality of small hive beetle adults and evidence of reproduction (visible larvae) was observed and recorded at 4-, 6-, 18-, and 34-day time points; cages with complete mortality were removed early.

### Field Trial

We trialed treated and untreated protein feeding in colonies in the last week of February 2022, when colonies were being managed to build up for springtime splits. Two batches of 4 kg BeePro (Mann Lake) protein supplement were weighed out. We added 200-ml mixtures, made of 80% glycerol, 20% methanol (v/v) to each batch; 1 mixture used methanol with 40 mg total chlorantraniliprole dissolved into solution. Batches were mixed thoroughly in clean buckets using industrial paint mixers. Control and treated protein supplement batches were then shaped into circular, equally-sized patties of 375 g (±1g), yielding 10 control, and 10 treated patties, where treated patties contained approximately 9.5 μg/g chlorantraniliprole. Two apiary sites containing only queenright, healthy honeybee colonies were selected, and 10 patties (5 control and 5 treatment) placed in 10 colonies at site 1, and 10 patties (5 control and 5 treatment) placed in 10 colonies at site 2. Patties were placed on a paper towel resting on frames in the top box of each hive, and a spacer shim was placed between the top box and lid to give ample space for the patties. Patties were all placed on the bright, clear, warm day on February 23rd during an early and warm spring approximately 1–3 wk before planned colony splits.

Following a period of 5 days, patties were removed and brought back to the laboratory. Surfaces of patties were observed under magnification for the presence of larvae, and the mass of each remaining patty was weighed to record the amount of supplement consumed by colonies. What remained of the patties were then incubated at 30 °C for 14 days and were then observed again to confirm the presence or absence of small hive beetle larvae in each of the patties.

### Statistical Analysis

All analyses were undertaken in the statistical programming language R v.4.0.4 “Lost Library Book.” We provide all data and analysis as a Zenodo-archived GitHub repository (DOI: 10.5281/zenodo.8381427). Almost all analyses had binary response variables and so used generalized linear models or generalized linear mixed-models with a binomial error structure. In one case where consumed mass was the response variable, a Gaussian (normal) linear mixed model was used. We tested for significance of predictor terms in our (G)L(M)Ms using either type-I (GLMs) or type-III (GLMMs) ANOVAs (χ^2^ or *F*-statistic depending on context) using the “afex” package (Singmann et al. 2019) which wraps around the “lme4” package (Bates et al. 2015) with subsequent examination of effect sizes and directions using the “emmeans” package (Lenth 2019).

For all cage trials we included cage as a random effect, and in the case of adult *Apis mellifera* trials also included which colony the bees were taken from, with cage nested under colony as a random effect. For the final field trial data, we supplemented our mixed models with Fisher’s exact test. For all adult honey bee toxicity assays we included up to 3 predictor terms: we always included the dose of the test chemical (numerical predictor), whether the feed solution contained methanol (binary yes/no), in cases of a positive control, whether there was dimethoate in the feed solution (binary yes/no), and for 1 assay, whether there was depletion of all of the feed solution (a starvation pressure, binary yes/no). For small hive beetle laboratory assays we only included “Dose” as a fixed predictor; we chose to analyze time-series data separately at each time point as cages were removed after complete cohort death.

## Results

### Preliminary Experiments

Preliminary screening using a filtered solution of a chlorantraniliprole lawn-drench (GrubEx, Scotts) showed promise; plates doused with lawn-drench solution showed complete *A. tumida* larval death at significantly higher rates than plates doused with control solution (χ^2^_1,22_ = 10.4, *P* = 0.001); correspondingly, larvae on treated plates showed higher mortality rates than their control counterparts (χ^2^_1,22_ = 102, *P* < 0.001).

Passive exposure to chlorantraniliprole failed to show any mortality in adult *A. tumida*. Cohorts of adult *A. tumida* placed in petri dishes with 1 square inch of paper towel soaked in 1 ml of sugar solution containing 5 or 10 µg/ml of chlorantraniliprole showed no elevated mortality compared to controls over 96 h of observation (only 1 of 217 adults across all test and control treatments died); there was no indication of consumption of the test solution by the adult beetles in this assay.

### Laboratory Assays

**Fig. 1.**
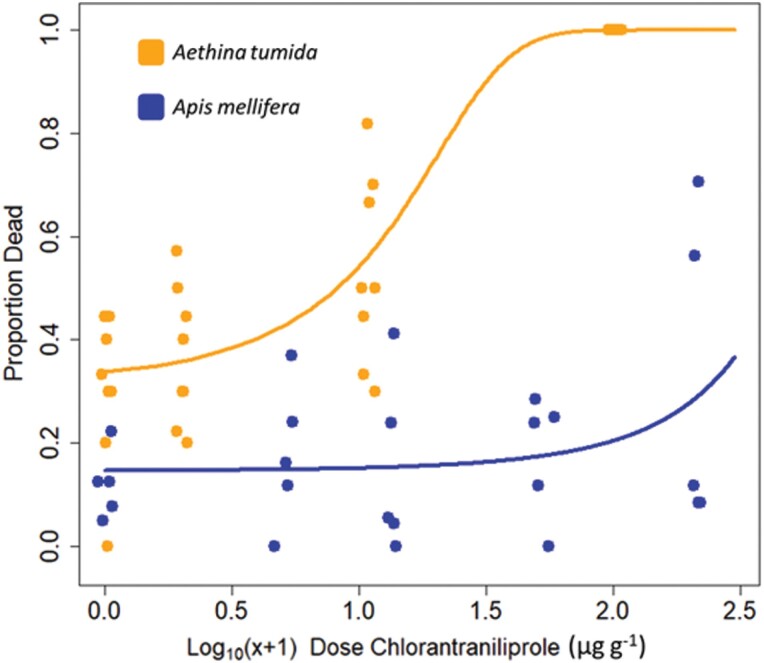
Dose–mortality curves for adult small hive beetles (orange upper line) and adult honey bees (blue lower line) orally exposed to chlorantraniliprole, via either incorporation into protein feed mix (small hive beetles) or sucrose solution (honey bees). Dose in µg/g is given on the x-axis, and proportion of individuals dead after 48 h is given on the y-axis. Note that we convert to a universal µg/g from the methodological µg/ml used for honey bee oral assays, where ρ = 1.23 for 1:1 w/w sucrose solution. A jitter is applied along the x-axis for easier interpretability and should not be interpreted as variation around the prescribed dose.

Oral toxicity of chlorantraniliprole to adult *A. tumida* was demonstrated when the test pesticide was incorporated into feed mix. Increasing concentrations of chlorantraniliprole correlated with higher rates of *A. tumida* mortality at every time point measured (Day 4: χ^2^_1,4_ = 67.3, *P* < 0.001; Day 6: χ^2^_1,4_ = 56.2, *P* < 0.001; Day 18: χ^2^_1,4_ = 47.8, *P* < 0.001); unsurprisingly therefore, higher concentrations of chlorantraniliprole also correlated with preventing reproduction (Day 18: χ^2^_1,30_ = 18.4, *P* < 0.001). Small hive beetles placed in reproductive cohorts on food mixtures with varied levels of chlorantraniliprole showed a characteristic dose–response amongst the adult beetles (Fig. 1), with complete mortality observed after less than 96 h at a concentration of 100 µg/g. Elevated adult mortality was observed at 10 µg/g, with no evidence of any successful reproduction at either of these concentrations. Initial evidence of reproduction (first or second instar larvae) was observed at 1 µg/g but larvae did not successfully develop to subsequent instars as was observed in the control (0 µg/g) food mixture.

Toxicity of chlorantraniliprole to honey bees was tested in adult *Apis mellifera* using oral feeding solutions. An initial test spanning chlorantraniliprole concentrations from 0 to 10 µg/ml showed no toxicity at 24 h (all adult honey bees in the experiment remained alive), and by 48 h feed solutions had been depleted, leading to starvation amongst some cohorts, with no evidence of any toxicity found across this dose span (χ^2^_1,6_ = 0.25, *P* = 0.615). The second, larger experiment incorporating a positive dimethoate control and excess feed solution (ad-libitum feeding) also showed no significant evidence of chlorantraniliprole concentration predicting mortality rates in adult honey bees (Fig. 1) after 48 h (χ^2^_1,6_ = 2.62, *P* = 0.106) whereas the positive dimethoate control showed complete mortality in under 24 h. We did anecdotally observe some lethargy amongst the cohorts feeding on the strongest chlorantraniliprole solutions (250 µg/ml), but observed no typical signs of acute pesticide poisoning or paralysis typically seen in honey bees such as trembling, shaking, stumbling, falling, or “drunk-like” behavior; rather, bees appeared to behave similarly to when chilled.

A similar assay using the alternative anthranilic diamide flubendiamide showed no evidence of toxicity up to 250 µg/ml (χ^2^_1,6_ = 0.01, *P* = 0.919) during ad-libitum feeding, with no observed secondary behavioral affects among any cohorts. Dimethoate positive controls showed complete cohort death within 24 h.

### Field Trial

**Fig. 2.**
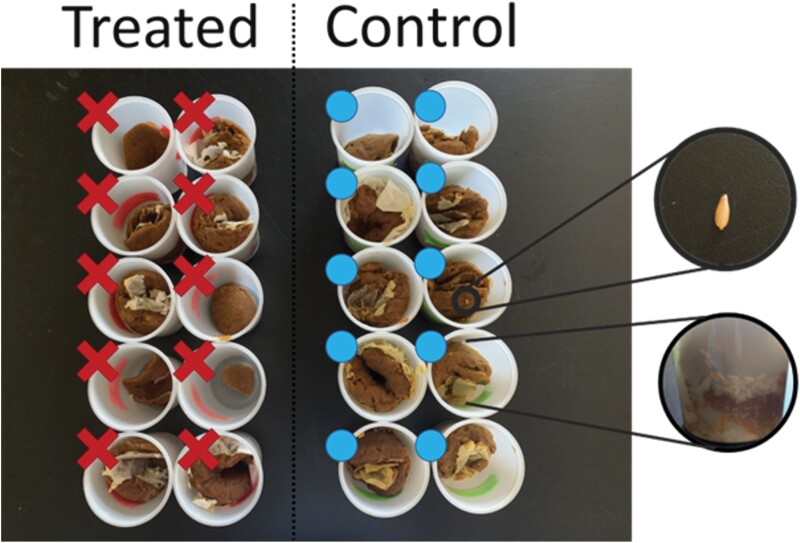
Presence or absence (blue dot or red cross respectively) of small hive beetle larvae in protein patties retrieved from the field after being placed in colonies for 4 days and subsequently incubated in the lab. Groups are separated into treated patties (left block of 10) and untreated patties (right block of 10). Experimental treatment perfectly mapped onto presence/absence of larvae, outset images show beetle larvae at various stages of development.

Following the in-lab trials focused on estimating the ranges of efficacy of chlorantraniliprole control of *A. tumida* and safety in *Apis mellifera*, we undertook field trials, comparing the outcomes of using treated and untreated controls when placed in springtime colonies (Fig. 2). We observed no significant difference in consumption rates of treated and untreated protein supplement patties by the honey bee colonies (*F*_1,8_ = 2.79, *P* = 0.112). Upon retrieval, 9/10 untreated (control) patties show signs of visible infestation (*A. tumida* larvae visible and characteristic “sliming” immediately beneath the patty inside the colonies) while none of the treated (test) patties showed either sign of infestation—a significant effect of treatment on apparent infestation (*F*_1,17.3_ = 81.0, *P* < 0.001). Following 14 days of incubation, 10/10 untreated patties hosted large numbers (hundreds) of *A. tumida* larvae. In contrast, all treated patties remained entirely free of infestation after 14 days of incubation in the same incubator; we can therefore conclude that treatment with chlorantraniliprole at this dose prevents small hive beetle reproduction in supplementary protein patties placed in honey bee colonies (Exact test: *P* < 0.001).

## Discussion

We show a profound difference in the toxicity of chlorantraniliprole to adult small hive beetles compared to adult honey bees (Fig. 1). Additionally, we show that chlorantraniliprole is capable of preventing small hive beetle infestation of apicultural protein mixes at concentrations which show no mortality to adult honey bees. We extend this finding to a field demonstration of its possible use (Fig. 2), whereby when chlorantraniliprole is used as an additive in supplemental protein for honey bees, it can completely prevent the threat of small hive beetle larvae developing in that protein feed, without impacting the proclivity of the honey bees to forage on the substitute supplemental protein mix. While further experiments are necessary, we believe that this is an extremely promising line of enquiry in the effort to develop better treatments for, and protection from, small hive beetle infestation in managed honey bee colonies.

We caution that additional work must be done to establish the safety of intentionally lacing honey bee feed with chlorantraniliprole and how this balances with the increased ability for beekeepers to feed their bees pollen patties or protein substitutes. Adults showed little to no additional mortality even at high doses in this experiment (Fig. 1) and others (Walker et al. 2022). However, direct assessment of the developing larvae exposed to chlorantraniliprole is warranted, as well as overall effects on colony health metrics as outlined in standard apicultural research (Berenbaum and Liao 2019). The abundance of work examining the action of chlorantraniliprole in honey bees, particularly Ricke et al. (2021) and Walker et al. (2022) which were motivated by documenting the possible damage chlorantraniliprole can cause to bee colonies when codeployed with other agricultural chemicals, such as fungicides, will be crucial to establishing its overall safety in beekeeping. Nevertheless, as stated these studies did inadvertently demonstrate the safety of chlorantraniliprole when deployed in isolation. The risk of co-exposure to environmental fungicides can only plausibly be assessed by trial deployment of the chemical in apiaries across the United States and in different agricultural contexts.

Two routes of deployment are foreseeable for chlorantraniliprole in honey bee colonies. The main focus of this manuscript has been as a feed additive to prevent the misappropriation of honey bee protein supplements by this destructive pest. The second, related use is the intentional baiting of small hive beetle traps with small quantities of highly-dosed protein feed, out of reach of honey bees (Kleckner et al. 2022). As established in this manuscript, the minimum preventative dose to be mixed into pollen patties is substantially lower than the lethal dose for adult small hive beetles. While a relatively low preventative dose is desirable to minimize any possible adverse effects on the honey bees, there are already on-market small hive beetle “traps” which feature apertures small enough to prevent honey bees from accessing the trap interior, but large enough to allow the entry of beetles. Placing small amounts of protein supplement mixed with much higher doses of chlorantraniliprole, at a concentration known to be lethal to the adult beetles, would be a safe way to also actively control the adult population without exposing honey bees to unnecessarily high chlorantraniliprole concentrations, especially if baits include an attractive volatile or pheromone mix (Suazo et al. 2003, Torto et al. 2005, Hayes et al. 2015, Stuhl 2021). Both the risk of residues in-hive products and concerns over possible accumulation in colonies will inform the use of these 2 deployments, and the migration of chlorantraniliprole into wax or other parts of colony will be a necessary topic of study prior to future use.

The promise shown by chlorantraniliprole in this study makes the prevention of small hive beetle invasion or expansion plausible in some regions, and extirpation of *A. tumida* in isolated pockets of beekeeping now seems more possible. However, the principal end goal of this initial foray into the use of chlorantraniliprole to control small hive beetles inside honey bee colonies is to allow for greater nutritional provisioning of managed honey bees in the United States and elsewhere. Increasingly, the multiplicative interactive effects of nutritional, toxicological, and parasite stress on honey bees is being highlighted as the major challenge to be surmounted by the industry. Chlorantraniliprole deployment for small hive beetle control could assist in the reduction of the parasite burden more broadly. Additionally, however, it allows for much easier and more economical feeding of honey bees, especially during early spring build up when colonies are being grown, nucleus colonies made, and package bees produced—although the availability of pollen locally is a large factor in determining the effectiveness of this (Noordyke and Ellis 2021). This effort is still however crucial to the wider US agricultural system, as the Southeast is one of the few regions where honey bee colonies can be brought up to adequate strength before being shipped to crucial pollination markets such as almonds, or sold to replace lost bees in more northerly reaches before the demands for pollination of crops such as apples. Larger, healthier honey bee colonies and less expensive replacement of lost winter colonies would reduce stress on multiple agricultural industries beyond beekeeping (Ferrier et al. 2018, Goodrich et al. 2019, Goodrich and Goodhue 2020).

Healthier honeybees arguably make for healthier wild bee populations by reducing the effects of spillover, especially of viruses (Manley et al. 2015, 2019). Any efforts to reduce viral burdens in the American honey bee stock, including by allowing for easier and more abundant provisioning of honey bee colonies with protein or pollen supplements (DeGrandi-Hoffman and Chen 2015, DeGrandi-Hoffman et al. 2016, Dolezal and Toth 2018), may therefore help the bee conservation agenda. Direct testing of whether chlorantraniliprole use would have this indirect antiviral effect on honey bees via improved nutritional provision is warranted. A further plausible benefit of this work is the suppression of *A. tumida* across the landscape. *Aethina tumida* are alleged to fly considerable distances (although this has yet to be decisively shown) and live upwards of 6 months (Roth et al. 2022), and currently have a suspected role in the parasitization and destruction of other, non-*Apis* bee colonies such as native *Bombus* species (Hoffmann et al. 2008). Suppression of their population and spillover potential may thusly prove important to bees beyond managed honey bees.
